# Supplementary material for: Persistent dynamic magnetic state in artificial honeycomb spin ice
Source: Nat Commun. 2023 Aug 25;14:5212. doi: 10.1038/s41467-023-41003-4 (PMC10457338; doi:10.1038/s41467-023-41003-4)
Supplement: Supplementary file 1 — Supplementary Information [file 41467_2023_41003_MOESM1_ESM.pdf]

Supplementary Information
Persistent dynamic magnetic state in artificial honeycomb spin ice

J. Guo[1,†], P. Ghosh[1,†], D. Hill[1,†], Y. Chen[2], L. Stingaciu[3], P. Zolnierczuk[3], C. A. Ullrich[1,*], and D. K. Singh[1,*]
[1] *Department of Physics and Astronomy, University of Missouri, Columbia, MO*
[2] *Suzhou Institute of Nano-Tech and Nano-Bionics, Chinese Academy of Sciences, China*
[3] *Oak Ridge National Laboratory, Oak Ridge, TN 37831, USA*
[†] *These authors contributed equally: J. Guo, P. Ghosh and D. Hill and*
[*] *Email: ullrichc@missouri.edu, singhdk@missouri.edu*

I. SUPPLEMENTARY METHODS

A. Nanofabrication of the artificial honeycomb lattice of permalloy

The fabrication of the artificial honeycomb lattice involves the synthesis of a porous hexagonal template on top of a silicon substrate, calibrated reactive ion etching to transfer the hexagonal pattern to the underlying silicon substrate and deposition of permalloy on top of the uniformly rotating substrate in a near-parallel configuration ($\sim 3°$) to achieve the 2D character of the system. The porous hexagonal template fabrication process utilizes diblock copolymer polystyrene (PS)-b-poly-4-vinly pyridine (P4VP) of molecular weight 29K Dalton and volume fractions 70% PS and 30% P4VP, which can self-assemble into hexagonal cylindrical structure of P4VP in the matrix of PS under the right condition. A PS-P4VP copolymer solution of mass fraction 0.6% in toluene was spin-coated on a polished silicon wafer (thickness $\sim$ 0.28 mm) at around 2300 rpm for 30 seconds, followed by solvent vapor annealing at $\sim 25°$C for 12 hours. A mixture of toluene/THF (20/80, volume fraction) was used for the solvent vapor annealing. This process results in the self-assembly of P4VP cylinders in a hexagonal pattern in a PS matrix. Submerging the sample in ethanol for 20 minutes releases the P4VP cylinders from the PS matrix, leaving a hexagonal porous template with an average hole center-to-center distance around 31 nm. The diblock template is used as a mask to transfer the topographical pattern to the underlying silicon substrate using reactive ion etching with CF_4 gas. The top surface of the etched silicon substrate resembles a honeycomb lattice pattern. Fig. S1 presents a depth profile along a cut line on an atomic force microscopic image of the honeycomb structure in the Si substrate. The depth of the honeycomb structure is on average around 5 nm. This honeycomb structure is then exploited to create a magnetic honeycomb lattice by depositing permalloy ($Ni_{0.81}Fe_{0.19}$) in a near-parallel configuration using E-beam physical vapor deposition. Samples were uniformed rotated to achieve uniformity of the deposition. This allowed evaporated permalloy to coat the top surface of the etched silicon substrate only and producing magnetic honeycomb lattice with a typical element size of about 11 nm (length) $\times$ 4 nm (width) and controllable thickness. A typical atomic force micrograph of the honeycomb lattice is shown in Fig. 1d. Samples with magnetic honeycomb layer of thickness 6 nm and 8.5 nm were used in this work.

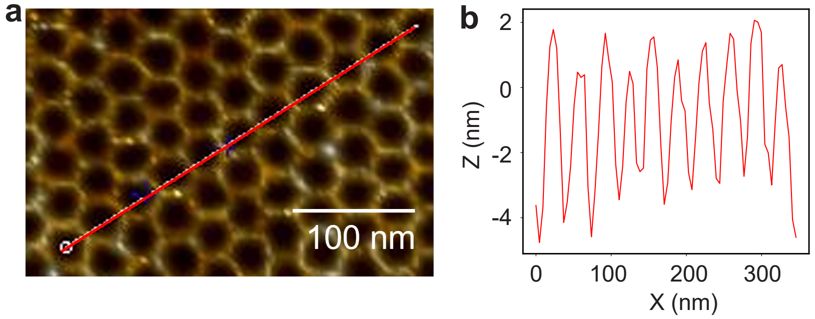

FIG. S1. **Characterization of the honeycomb structure depth in Si substrate. a** Atomic microscopic force image of the honeycomb structure in the Si substrate. **b** Depth profile Z vs X along the cut line in **a**. On average, the depth of the honeycomb structure is $\sim$ 5 nm. This honeycomb structure on the substrate is then exploited to create magnetic honeycomb lattice by depositing magnetic material in a near-parallel configuration.

B. Time-of-flight Neutron Spin Echo instrument

The Neutron Spin Echo (NSE) measurements were conducted on an ultrahigh resolution (2 neV) neutron spectrometer with a dynamic range 1 ps to 300 ns at beam line BL–15 of the Spallation Neutron Source, Oak Ridge National Laboratory. A 3 Å wavelength bandwidth of neutrons (3.5–6.5 Å) was used in the experiment, and the scattered neutrons were collected with a 30 cm $\times$ 30 cm position-sensitive ^{3}He detector 3.9 m away from the sample. The detector has 32 $\times$ 32 X, Y pixels laterally to keep track of the scattering angles of the detected neutrons. It also has 42 time of flight (ToF) channels (tbins) that label the time frame of the detected neutron which encodes the detected neutron's wavelength. Thus, the data collected by the detector is a 32 $\times$ 32 $\times$ 42 array. By carefully grouping the X, Y pixels and tbins, one can extract echo signals at different Fourier times t [see Eq. (S2) below for the definition] and q values from a single echo measurement. Note that for a 2D area detector with ToF, each detector pixel corresponds to a broad range of q arising from the wavelength band of neutrons from the source. In this sense, the 42 ToF tbins essentially define the resolution of the wavelength of the detected neutrons. Suffice to say that at a given X, Y detector pixel, within a single tbin, the collected neutrons are assumed to have the same wavelength, hence the same q and Fourier time t. The scattering vector q also varies a cross to the detector area due to the change of scattering angle. For illustration purposes, in Fig. S5, we have presented the q-assignment of the detector pixels within a single tbin corresponding to a Fourier time $t = 0.02$ ns.

Unlike in the soft matter NSE where the echo signal and the normalization factor can be measured in the same instrument configuration, paramagnetic NSE requires separate measurements for the echo signal and the total magnetic scattering for the normalization purpose. In the echo signal measurement, the scattered beam intensity is measured as one scan through the phase current that introduces the field integral asymmetry, and no field is applied around the sample. Note that in paramagnetic NSE, instead of using a π flipper before or after the sample to flip the neutron's polarization, the magnetic sample itself does a π flip of the neutron's polarization due to the nature of the interactions between magnetic moments and neutron's polarization. Thus, in this experiment, the π flipper was removed in the echo signal measurement. This way, we are certain that any scattering effects that cannot invoke a π flip to the neutrons' polarization (including structural scattering) will not contribute to an echo signal but only a flat background [1]. In this experiment, echo signals at multiple temperatures and nominal Fourier times (the Fourier time associated with the neutron of the maximum wavelength) were measured.

The total magnetic scattering is measured in the 3D xyz polarization analysis where a small guide field is applied around the sample. For this purpose, three sets of Helmholtz-style coils were orthogonally mounted around the sample position for x, y and z orientations, see Fig. S2 for details of the coil installment. Six additional polarization measurements along x, y and z directions $(x_{\rm up}, x_{\rm dn}, y_{\rm up}, y_{\rm dn}, z_{\rm up}, z_{\rm dn})$ were performed at each temperature with a small guide field $\sim$ 10 Oe applied around the sample to define the quantization direction for the neutron spin and maintain its polarization. A magnetic needle was used to make sure the polarization field direction aligns to the x, y and z orientations. Here, $z_{\rm up}$ and $z_{\rm dn}$ for instance, were measured as the spin-non-flip scattering (SNF) and the spin-flip scattering (SF) cross sections with the field along the positive z direction. The polarization analysis was performed according to the method described by Pappas et al. [1] with the currents in the coils tuned following Ehlers et al. [2]. Note that considering the strong exchange coupling between the magnetic moments in the sample, we believe that the small field of 10 Oe is very unlikely to affect the magnetization or bias the domain in our honeycomb lattice during the xyz polarization analysis, just like in other neutron measurements that utilize polarized neutrons. It also does not affect the relaxation dynamics in the sample since the relaxation events are probed using echo signal measurement, which does not employ the guide field around the sample. Domain wall formation is also not possible since the size of honeycomb element ($\sim$ 11 nm) is a bit smaller than the typical permalloy domain size ($\sim$ 12 nm).

To obtain good signal-to-background ratio, a stack of 125 (117) samples of 6 nm (8.5nm) of about 20 $\times$ 20 mm^2 was loaded in a custom-made aluminum sample container. The Si substrate for each sample is $\sim$ 0.28 mm in thickness. Thus, considering the macroscopic separation distance between the magnetic honeycomb layers, we believe that there should be no inter-layer couplings between the magnetic honeycomb layers. The sample container was then inserted into a close cycle refrigerator with a base temperature of 4 K with the sample stack exposed to the neutron beam in the transmission geometry such that the neutron beam direction is parallel to the sample normal direction. A schematic diagram of the modified NSE instrument is presented in Fig. S3.

C. Analysis of the Neutron Spin Echo signal

NSE experiments probe the intermediate scattering function of the sample studied, given by [3]

$$S(q,t) = \int \cos(\omega t) S(q,\omega) d\omega, \qquad \text{(S1)}$$

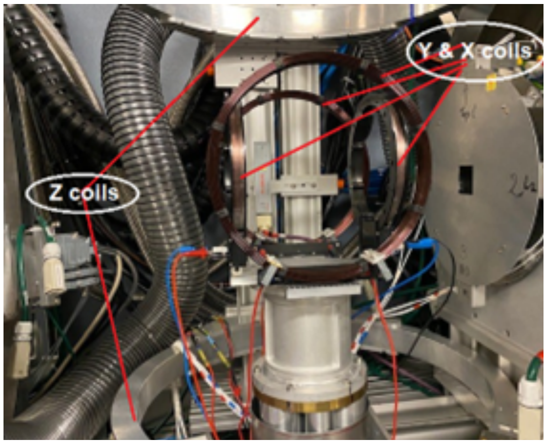

FIG. S2. **The set-up of the magnetic coils in the polarization analysis.** The three coils are orthogonally mounted in the x, y and z directions.

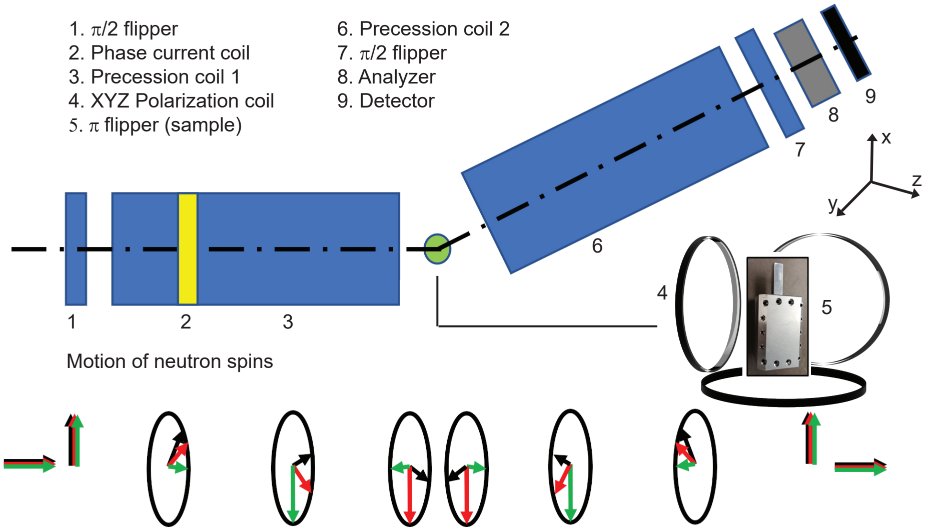

FIG. S3. **Design of modified NSE instrument, utilized for the magnetic charge relaxation experiment.** Inset shows the stack of samples sealed in an aluminum can, which serves as a π flipper. Additional magnetic coils are installed to enable the polarization analysis.

where $S(Q,\omega)$ is the scattering function of the sample and t is the Fourier time, defined as

$$t = J\lambda^3 \frac{\gamma_\mathrm{n} m_\mathrm{n}^2}{2\pi h^2}, \tag{S2}$$

with γ_n, m_n and λ being the neutron's gyromagnetic ratio, mass and wavelength, and h denotes Planck's constant. $J = \int |B| dl$ is the magnetic field integral along the neutron's path through the precession coil [4]. The field integrals are designed to be the same in both precession coils before and after the sample such that the precession phase acquired by the neutron in the first coil will be exactly recovered at the end of the second coil, given that the neutron does not change its velocity while interacting with the sample. For quasi-elastic scattering, the neutron gains a net phase at the end of the second coil before it reaches the analyzer. By systematically stepping through an additional phase current (convert to an additional field integral) in either the first or the second coil, a cosine modulation of the sample's intermediate scattering function is realized, which is the typical raw data, echo signal, one would analyze in an NSE experiment.

The echo signal intensity for a given neutron wavelength λ has a cosine function shape given by

$$I(\phi) = A\cos(\phi\lambda) + B, \tag{S3}$$

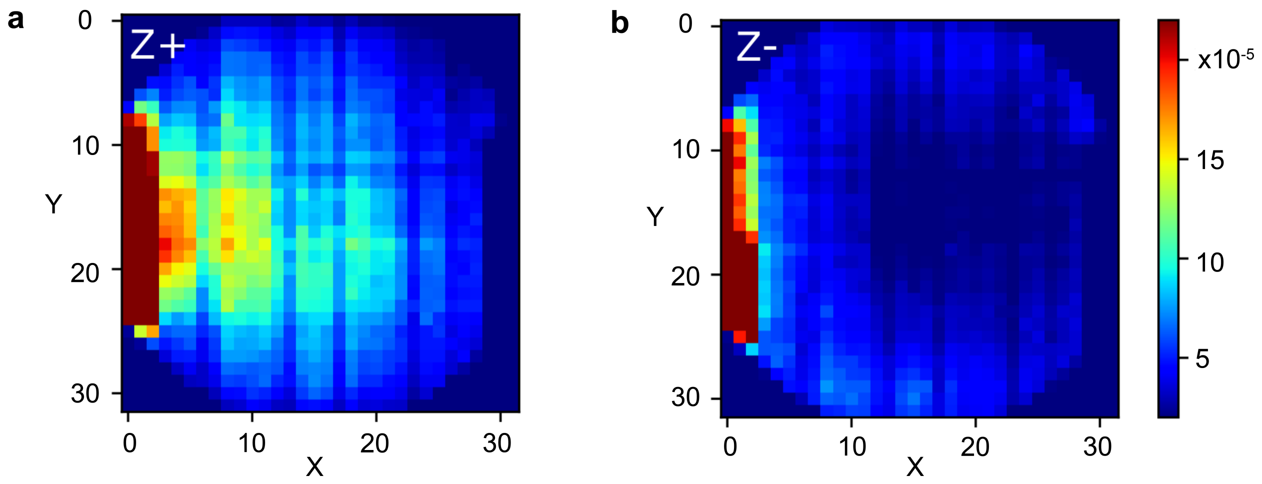

FIG. S4. **Color detector image of the 6nm honeycomb lattice at room temperature. a** Detector image with neutron polarization Z+. **b** Detector image with neutron polarization Z−. The color scale on the right shows the detected neutron intensity normalized by the proton charge. With the same color scale, while no meaningful spectral weight is detected in the Z− polarization, in the Z+ polarization case, clear spectral weight is observed in the detector region corresponding to $q \sim 0.058$ Å^{-1} and $q \sim 0.029$ Å^{-1}. The intense signal at the left edge of the detector is the direct beam as the instrument hits its geometrical constraint limit. Fig. 2a in the main text is obtained by subtracting the Z− intensities from the Z+ intensities.

where $\phi = dJ\gamma_n m_n/h$ and dJ is the phase asymmetry between the two precession coils introduced via the scanning phase current. When signals from neutrons of a certain wavelength span $\lambda_{avg} \pm d\lambda$ are added up, the total intensity resembles a cosine function modulated by an envelope function

$$I(\phi) = A\cos(\phi\lambda_{avg})\frac{\sin(\phi d\lambda)}{\phi d\lambda} + B. \tag{S4}$$

II. SUPPLEMENTARY NOTES

A. Neutron Spin Echo raw data treatment

The echo intensities were obtained by adding up signals in X, Y detector pixels and then summing over different ToF tbins. Grouping of the X, Y detector pixels were guided by the false color detector image (Fig. 2a in the main text) as well as the detector pixel image (Fig. S5) to center around the intense scattering signal.

For the echo profiles presented in Fig. 2 in the main text, data from a single ToF tbin is exploited with the phase shift along Y direction corrected for demonstration purpose. For the 8.5 nm sample, the echo profiles (Fig. 2c,f in the main text) were obtained by summing over detector area of 3 × 12 in X and Y directions (X = 8 to 10, Y = 10 to 21) with the mean neutron wavelength 3.7 Å in an effective wavelength band of 0.07 Å. In order to make direct comparisons between the 6 nm honeycomb lattice and the 8.5 nm honeycomb lattice, echo profiles of the 6 nm sample (Fig. 2b,d,e of the main text) with the same Fourier time $t = 0.02$ ns are presented. They were obtained by summing over detector area of 5 × 18 in X and Y directions (X = 7 to 11, Y = 7 to 24) with the mean neutron wavelength of 4.6 Å in an effective wavelength band of 0.07 Å. Note that a slightly larger detector area was selected for the data analysis of the 6 nm sample. This is because the detected scattering signal from the 6 nm sample is broader than that from the 8.5 nm sample, possibly due to a larger variation of the honeycomb element length scale. We present in Fig. S6 echo profiles of the 6 nm sample measured at room temperature with increasing Fourier time t. Together with Fig. 2b,d, it is clear that as the Fourier time increases, the error bars become large and the echoes exhibit smaller amplitudes. Fig. S7 shows the echo profiles of the 6 nm sample with the same Fourier time $t = 0.02$ ns, measured at different temperatures. One can see that good signal to background ratio is persistent from room temperature to the base temperature 4 K at low Fourier time values. In Fig. S8, we show the echo profiles at an intermediate temperature $T = 50$ K with Fourier time $t = 0.02$ ns, as well as echo profiles at higher Fourier times. Also note that, the echo profiles presented in Fig. 2 in the main text as well as Fig. S6, Fig. S7 and Fig. S8 are raw data without

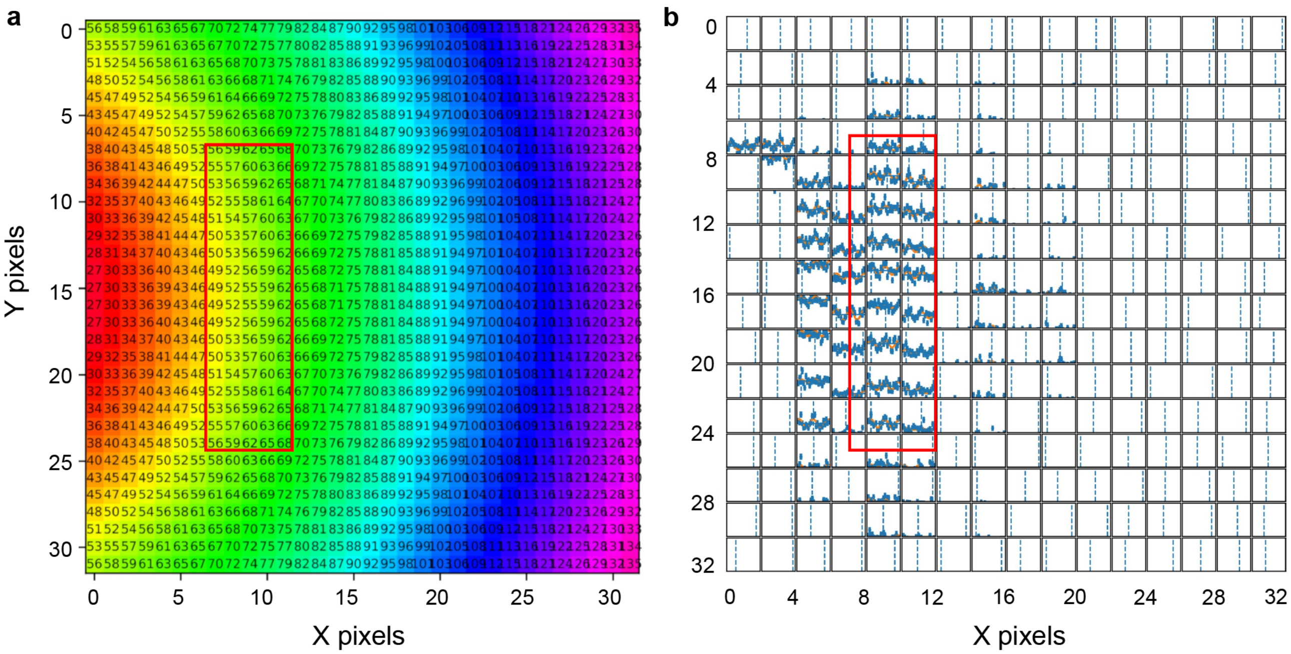

FIG. S5. **The q distribution and echo signals of the 6 nm honeycomb lattice measured at T = 4 K on the detector. a** The flux-weighted q distribution across the whole detector in ToF tbin 17, corresponding to a Fourier time of 0.02 ns, the numbers in the individual pixels denote the q value in the unit of 0.001 Å^{-1}. **b** The echo signals in ToF tbin 17 on the detector. Every 2 pixels along X and Y directions are grouped for the clearness of the demonstration. The intense direct beam signals on the left edge of the detector are not shown. This image together with the color detector image in Fig. 2a in the main text guided the grouping of detector X, Y pixels in data analysis.

normalization to the neutron flux. For this reason, average intensities from echo to echo might vary due to differences in neutron counts acquisition time or neutron flux surges.

For the calculation of the intermediate scattering functions (Fig. 3a,b of the main text and Fig. S10) at each temperature, identical X, Y pixel groupings used for the echo profiles are directly summed up. At each temperature, independent echo measurements were performed at 5 nominal Fourier times (0.06 ns, 0.1 ns, 0.3 ns, 0.7 ns, 1.0 ns) by set different values for the field integral J. From each echo measurement at a given nominal Fourier time, 3 echo signals of different Fourier times were extracted by grouping ToF tbins, T = 10 to 19, T = 20 to 29, T = 30 to 39, respectively, with the phase shift along the tbins corrected. These echo signals were normalized to the neutron flux and were further analyzed for the intermediate scattering function calculation.

B. Calculation of the intermediate scattering function

The goal of every NSE experiment is to obtain the intermediate scattering function $S(q,t)/S(q,0)$ as a function of the Fourier time t, which can be calculated from the fitted echo amplitude. For non-magnetic samples,

$$\frac{S(q,t)}{S(q,0)} = \frac{2A}{U-D} \tag{S5}$$

where A denotes the fitted amplitude of the echo signal, U and D denote the spin up (non-π flipping) and spin down (π flipping) measurement of direct scattering without precession. $U-D$ measures the maximum obtainable echo amplitude and is used as the normalization factor. In paramagnetic NSE with a magnetic sample, half of the magnetic scattering intensity $M/2$ is used for normalization [1]. M and U, D are calculated as

$$M = 2(z_{\mathrm{up}} - z_{\mathrm{dn}}) - [(x_{\mathrm{up}} - x_{\mathrm{dn}}) + (y_{\mathrm{up}} - y_{\mathrm{dn}})], \tag{S6}$$

$$U = \frac{x_{\mathrm{up}} + y_{\mathrm{up}} + z_{\mathrm{up}}}{3}, D = \frac{x_{\mathrm{dn}} + y_{\mathrm{dn}} + z_{\mathrm{dn}}}{3}. \tag{S7}$$

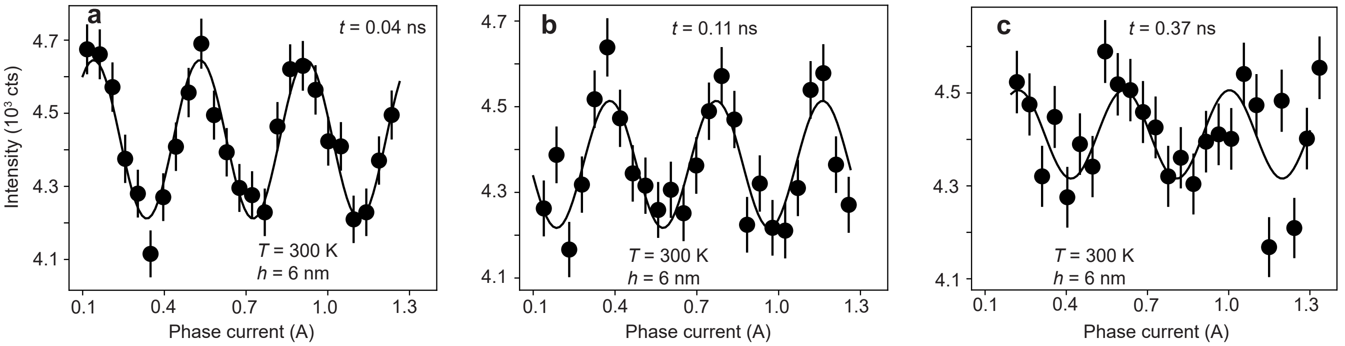

FIG. S6. **Echo profiles of the $h = 6$ nm honeycomb lattice at different Fourier times measured at room temperature.** **a** $t = 0.04$ ns. **b** $t = 0.11$ ns. **c** $t = 0.37$ ns. The Fourier time of the echoes are labeled on the graphs. Clearly, as the Fourier time increases, the error bars become large and the echoes exhibit smaller amplitudes. In all plots, the error bar represents one standard deviation.

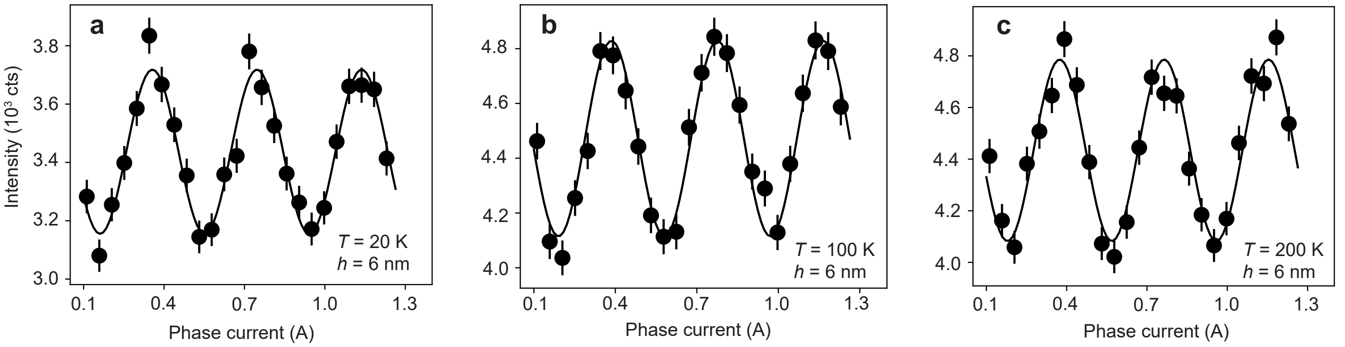

FIG. S7. **Echo profiles of the $h = 6$ nm honeycomb lattice at Fourier time 0.02 ns measured at intermediate temperatures.** **a** $T = 20$ K. **b** $T = 100$ K. **c** $T = 200$ K. Put together with Fig. 2b,e of the main text, the persistence of a good signal-to-background ratio from room temperature to the lowest measurement temperature (4 K) demonstrates the high quality of the data. In all plots, the error bar represents one standard deviation.

Therefore, one way of estimating the signal to background ratio is to calculate $M/(U + D)$, which is the contribution of magnetic scattering to the total scattering. For instance, the signal to background ratio is estimated to be $\sim 3.4\%$ at $T = 4$ K, and $\sim 3.5\%$ at $T = 300$ K for the 6 nm sample, which is not surprising considering the small mass of the sample investigated. In the meantime, in paramagnetic NSE, only magnetic scattering creates an echo signal without a π flipper at the sample (See Fig. 2, Fig. S6, Fig. S7). Thus, one can calculate A/B fitted from Eq. (S4) as another indication of the signal to background ratio. The intermediate scattering function is then determined as

$$\frac{S(q,t)}{S(q,0)} = \frac{4A}{M}, \tag{S8}$$

which is comparable with $66A/(U - D)$ in this experiment. In the calculation of the intermediate scattering function at a given Fourier time, the same X, Y pixels and ToF tbins selection was used for the determination of both the echo amplitude and the normalization factor.

Unlike experiments conducted with other spectrometers, the measured dynamic structure factor $S_{\mathrm{exp}}(q,\omega)$ is a convolution between the true dynamic structure factor $S(q,\omega)$ and the instrument resolution $R(q,\omega)$, that is $S_{\mathrm{exp}}(q,\omega) = S(q,\omega) * R(q,\omega)$. NSE directly probes the intermediate scattering function, which is the Fourier transform of the dynamics scattering function $S(q,t) = \int \cos(\omega t) S(q,\omega) d\omega$, thus $S_{\mathrm{exp}}(q,t) = S(q,t)R(q,t)$. Therefore, in an NSE experiment, the resolution function can be simply divided out to obtain the true intermediate scattering function. At SNS-NSE the resolution of the instrument and elastic scattering contribution is assessed by measuring a perfect elastic scattering sample, usually mounted in the same container as the sample to investigate, measured over the same q range, Fourier time range, and wavelength. For soft matter measurements, solid graphite and Al_2O_3 as well as TiZr are usually used depending on the scattering angles. For paramagnetic NSE measurements, $Ho_2Ti_2O_7$, a well-known classical spin ice material frozen below $T = 20$ K is used where it exhibits no dynamics. However, for

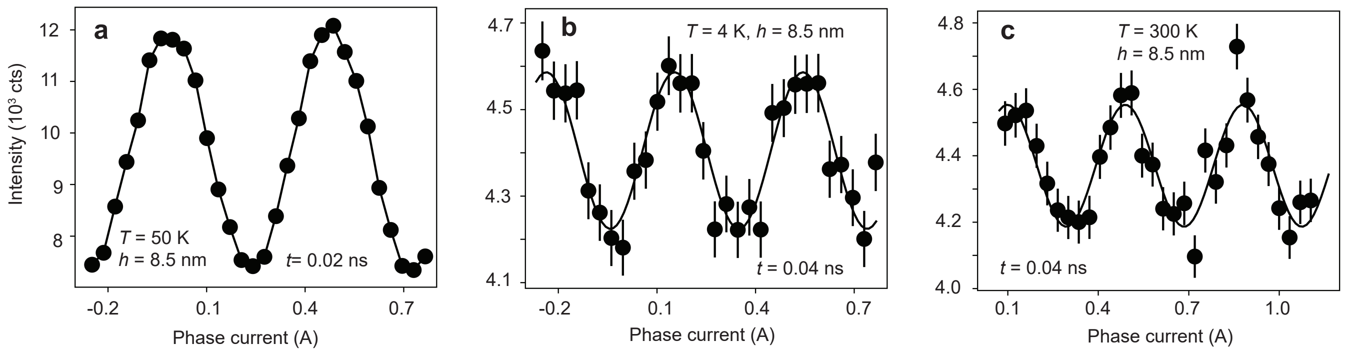

FIG. S8. **Echo profiles of the $h = 8.5$ nm honeycomb lattice measured at intermediate different temperatures and Fourier times.** **a** Echo profile at $T = 50$ K with $t = 0.02$ ns. **b** Echo profile at $T = 4$ K with $t = 0.04$ ns. **c** Echo profile at $T = 300$ K with $t = 0.04$ ns. The phase difference between the echoes is caused by the difference in the respective Fourier time t. In all plots, the error bar represents one standard deviation.

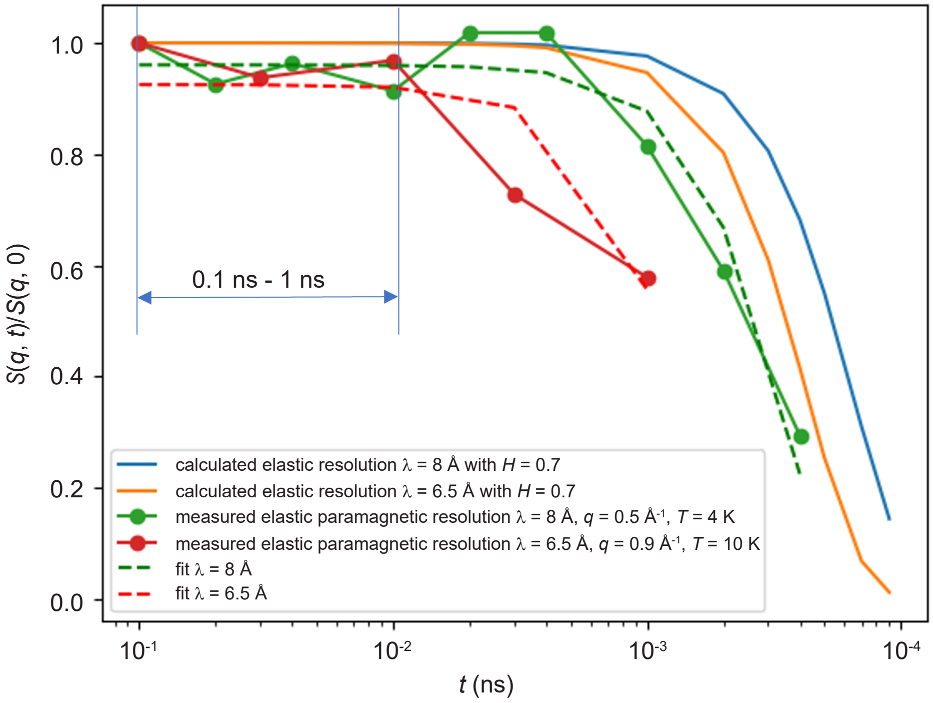

FIG. S9. **Measured paramagnetic resolution function at SNS-NSE and simulated elastic resolution function in soft matter regime.** The paramagnetic resolution functions are measured at different q values on a $Ho_2Ti_2O_7$ sample at temperatures below its frozen temperature $T = 20$ K, with two different neutron wavelengths. H represents the relative field integral homogeneity of the SNS-NSE spectrometer, a calculated value specific for SNS-NSE. Clearly, the paramagnetic resolution function below Fourier time $t < 1$ ns is predominantly flat. Thus, any resolution correction to the measured intermediate scattering function in this work will not affect the relaxation time extracted from the exponential fitting, but only shift the data on the y-axis.

the measurement performed in this work, it was not possible to use $Ho_2Ti_2O_7$ sample as the resolution since it only scatters and produces reliable echoes at high scattering angles, while the magnetic honeycomb samples scatter in the small-angle regime. Another common practice in quasi-elastic techniques is to measure the same sample at $T \leq 4$ K where presumably all dynamics freezes. However, it is well observable in our measurements that even at $T = 4$ K fast dynamics of the magnetic charges still exists, which is, in fact, one of the main findings of our research. Therefore, we decided not to reduce the data by elastic resolution, and the relaxation times τ_m were extracted directly from fitting the intermediate scattering function at each temperature, since, as explained, we do not have an elastic scatter with

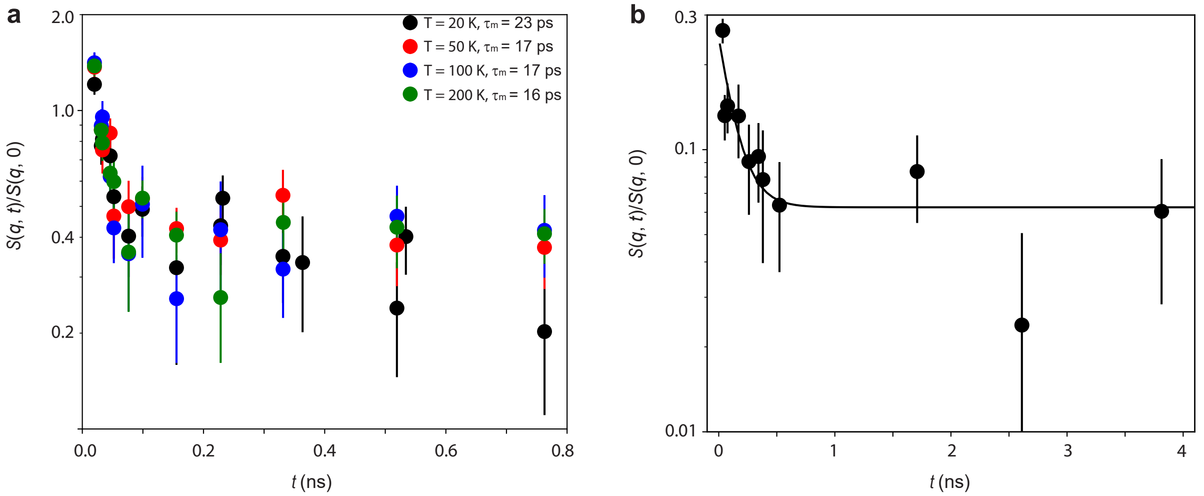

FIG. S10. **Normalized intermediate scattering function.** **a** Normalized intermediate scattering function of the 6 nm honeycomb lattice at $q \sim 0.058$ Å^{-1} at intermediate temperatures, related to Fig. 3 of the main text. The relaxation time τ_{m} at each temperature are indicated on the graph. **b** Normalized intermediate scattering function of the 8.5 nm honeycomb lattice at $q \sim 0.06$ Å^{-1} at $T = 4$ K, related to Fig. 3 in the main text. In both plots, the error bar is calculated from the square root of the variance of the least-square-fitted parameter.

frozen dynamics within the temperature range accessible. In support of our decision is the fact that at SNS-NSE below Fourier times $t < 1$ ns, the elastic resolution is predominantly flat (linear). This means that any reduction by resolution will not affect the relaxation observed in the data but only the intensity scaling on y-axis. To demonstrate the aforementioned feature of the elastic resolution, we have collected two measured $Ho_2Ti_2O_7$ resolutions from previous paramagnetic measurements at $T = 4$ K and 10 K, for 2 different wavelength 6.5 Å and 8 Å. In Fig. S9, these experimental elastic resolution magnetic data are presented, together with their fits and simulated resolution in the soft-matter regime. One can easily observe the linear behavior in the range of $t < 1$ ns, which is the predominant range of our measurements. In this sense, the conclusion of a thermally independent quantum mechanical relaxation process that persists below the defects' activation energy in the 6 nm honeycomb sampe will still be valid with instrument resolution correction since the correction equally applies to each temperature. Plots of $S(q,t)/S(q,0)$ at $q = 0.058$ Å^{-1} at different temperatures are presented at Fig. 3a,b in the main text as well as Fig. S10. Note that exact values of $S(q,t)/S(q,0)$ in Fig. 3a, b and Fig. S10 are in arbitrary unit and are not suitable for comparison, as they are not instrument resolution corrected. In addition, they are calculated for samples with different overall mass that were measured at different times. It is the estimation of the relaxation time from $S(q,t)/S(q,0)$ that is of the most scientific importance. While the error bar of $S(q,t)/S(q,0)$ increases as the Fourier time increases as can be also inferred from the increasingly noise echo signal shown in Fig. S6, the error bar is relatively smaller in low Fourier time regime where it is more important for the estimation of relaxation time.

C. Theoretical details

Here we present a theoretical analysis of the ultra-small artificial magnetic honeycomb lattice, with the intention of elucidating the dominant physics at play behind the experimentally observed temperature independence of the magnetic charge relaxation time.

As discussed in the main text, we suspect the system exhibits rapid renormalization group (RG) convergence to a minimal effective model. This intuition is motivated by the simple, consistent behavior of the magnetic charge relaxation over a broad range of temperatures; a feature that is unchanged with at least a modest change in lattice thickness. By "rapid renormalization group convergence" we specifically mean that small changes to a hypothetical exact theoretical description of the experimental system, such as a change in an interaction term coupling strength or a change in system size, would result in no qualitative change to the physically observable behavior of the model, due to the presence of a well isolated and strongly stable fixed point in RG space. Literature on the structurally equivalent Kagome spin ice model supports the contention that the model exhibits rapid RG convergence to the same high and low temperature phases, with or without the inclusion of long or short range interaction terms. [5] As such we assume the system should be modeled effectively by a Hamiltonian with minimal features, e.g. a Hamiltonian

that only captures the magnetic charge excitation energy and the dynamical capacity of these charges to transfer to neighboring sites.

In the following subsections, we consider two minimal effective models, one a classical description and one a quantum mechanical description, and compare the excitation relaxation time for each model to the experimental results.

1. *Quantum magnetic charge model*

The simplest model for a Kagome spin ice involves spins

$$\mathbf{S}_i = \sigma_i \hat{\mathbf{e}}_i, \tag{S9}$$

restricted to specific site dependent directions $\hat{\mathbf{e}}_i$ which point along three possible axes separated by 120°. These axes together form a honeycomb lattice structure akin to the herein studied artificial honeycomb lattices. The spins of the Kagome spin ice are coupled via a nearest-neighbor spin exchange interaction

$$H_1 = -J_1 \sum_{\langle i,j \rangle} \mathbf{S}_i \cdot \mathbf{S}_j = \frac{J_1}{2} \sum_{\langle i,j \rangle} \sigma_i \sigma_j, \tag{S10}$$

where $\hat{\mathbf{e}}_i \cdot \hat{\mathbf{e}}_i = -\frac{1}{2}$ has been used, and the notation $\langle \ldots \rangle$ is used to indicate a sum over nearest neighbors. The system is frustrated in the case of ferromagnetic coupling, i.e. $J_1 > 0$, as can be seen by noting that for this sign of J_1 the far right side of Equation (S10) is equivalent to an antiferromagnetic honeycomb Ising model. The Hamiltonian (S10) can be simplified further by introducing the magnetic charge at honeycomb vertex α, $Q_\alpha = \pm \sum_{i \in \alpha} \sigma_i$. In terms of these operators, the Hamiltonian is equivalent to, up to a constant,

$$H_1 = \frac{J_1}{4} \sum_{\alpha} Q_\alpha^2. \tag{S11}$$

Here the possible values of the magnetic charges are $Q_\alpha = \pm 1, \pm 3$, with the ± 3 states corresponding to the localized excitations of the model.

The model described by H_1 is essentially classical, for the same reason that the original Ising model (without a transverse field) is classical, namely because the model lacks any non-commuting observables. This originates in the fact that, in deriving this Hamiltonian, we have neglected the important off axis components of the spins in Eq. (S9). Including these off axis terms in the exchange coupling of Equation (S10) results in nearest neighbor spin flip-flop terms (corresponding to a nearest neighbor hop of Q_α), thus restoring the quantum nature of the model. However, instead of simply reintroducing the spin components, we consider a slightly different starting point in hopes of constructing a minimal quantum mechanical model in terms of the Q_α. To this end, we note that the quartet nature of Q_α suggests that the simplest possible treatment of Q_α as a quantum operator would be to treat it as a z component of a pseudospin 3/2 representation of SU(2). With this in mind the natural choice of non-commuting observables would be the ladder operators of the same pseudospin 3/2 representation. With a basic nearest neighbor hopping term, we arrive at the minimal quantum mechanical Hamiltonian

$$H_Q = J_1 \sum_{\alpha} q_\alpha^2 + J_\mathrm{h} \sum_{\langle \alpha,\beta \rangle} q_\alpha^+ q_\beta^-, \tag{S12}$$

where we define $q_\alpha = \frac{1}{2} Q_\alpha$ in order for q_α to exhibit the traditional spin 3/2 representation eigenvalues of $\pm\frac{1}{2}$ and $\pm\frac{3}{2}$, and the ladder operators $q_\alpha^\pm$ are defined by their SU(2) commutation relations

$$\left[q_\alpha, q_\beta^\pm\right] = \pm q_\alpha^\pm \, \delta_{\alpha\beta}, \quad \left[q_\alpha^+, q_\beta^-\right] = 2 q_\alpha \, \delta_{\alpha\beta}. \tag{S13}$$

In order to model excitation relaxation time using the Hamiltonian H_Q, we consider two separate approximation schemes, a fully integrable finite size model with 4 sites, and a dynamic Monte Carlo algorithm applied to an extended periodic boundary condition model with 200 sites.

For the first approximation, using the short range nature of the Hamiltonian, we focus on a central vertex coupled to three isolated neighbors via the flip flop term of Eq. (S12). The result is a 4^4 dimensional Hilbert space system which we time evolve exactly. For the initial ensemble, the system is set at thermal equilibrium and projected into only states with a central q eigenvalue of $q_0 = +\frac{3}{2}$. We calculate the central excitation probability $P(q_0 = +\frac{3}{2})$ as a function of time. The resulting time dependence of the $q_0 = +\frac{3}{2}$ state shows a temperature independent relaxation

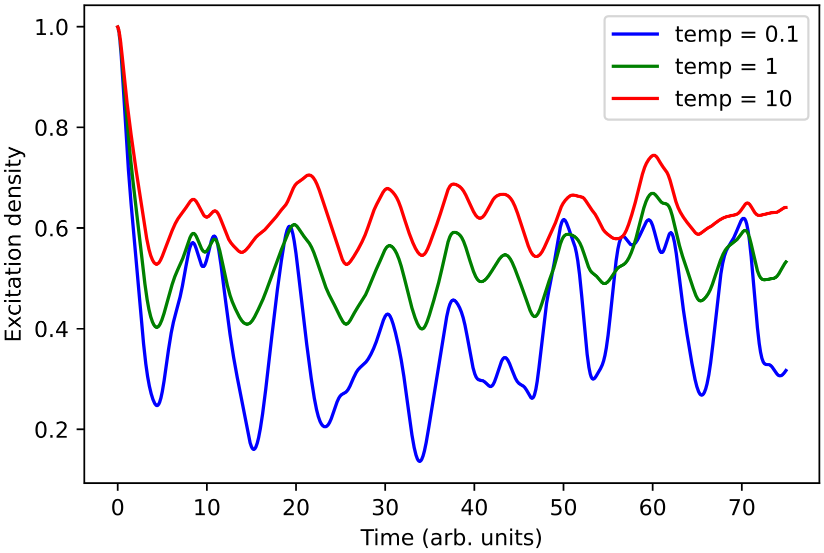

FIG. S11. **Exact time evolution of a four site quantum spin ice model ensemble with a central $Q = 3$ excitation initial state.** The curves show the density of the central charge over time for three different initial temperatures of the surrounding sites.

time, in agreement with experimental results. The main qualitative change as a function of temperature is that the large time asymptote of the time average of $P(q_0 = +\frac{3}{2})$ increases monotonically with temperature, as one would expect from ergodicity. These features can be seen in Fig. S11.

For the second approximate treatment of Equation (S12), we utilize the dynamic Monte Carlo algorithm as presented in Ref. [6]. The system we consider has dimensions of 10 unit cells by 10 unit cells, with 2 sites per unit cell, for a total of 200 sites, under periodic boundary conditions. We time evolve the system according to the dynamic Monte Carlo algorithm through thousands of hopping processes, recording any excitations that appear in the system and computing the average lifetime of stationary excitations. In this case, it is straightforward to model the system with the addition of a potential energy barrier to magnetic charge hopping. The experimental result of temperature independent relaxation time is reproduced for the case of this barrier being set to zero, as shown in Fig. 4b. Given the fact that exchange energy is expected to be much larger than the estimated barrier height, ~ 20 K (as discussed below), the magnetic charge relaxation between neighboring vertices can be considered barrier-free.

2. *Classical spin dynamics model*

The large number of spins per honeycomb joint in the experiment, by conventional wisdom, would suggest the possibility of modeling the system according to the purely classical treatment of a Landau-Lifshitz-Gilbert (LLG) equation of motion. Here we provide such treatment while following the same philosophy as that above of restricting ourselves to a minimalistic model. As the starting point for the above kagome spin ice was the nearest neighbor spin exchange coupling, we start with the classical analogue of this term in our LLG Hamiltonian. We further posit an anisotropy term in order to favor spins pointing along the honeycomb joint axes described by the $\hat{\mathbf{e}}_i$ unit vectors. The result is the Hamiltonian

$$H_{\mathrm{LLG}} = -J \sum_{\langle i,j \rangle} \mathbf{S}_i \cdot \mathbf{S}_j + K_1 \sum_i \left(\mathbf{S}_i \cdot \hat{\mathbf{e}}_i \right)^2 , \tag{S14}$$

where $\mathbf{S}_i$ are normalized classical spin variables. Physically a term like K_1 arises in an effective field theory typically with dominant contributions from shape anisotropy; however this term can be used to wrap up a variety of both intrinsic and emergent anisotropic effects all into a single, phenomenological parameter. As such K_1's optimal value can potentially be difficult to estimate both experimentally and theoretically. However, we have used the typical value for permalloy, $K_1 \sim -1 \times 10^4$ J/m^3.

In order to model the effects of temperature, we include a time dependent stochastic field $\mathbf{B}_{\rm th}$ in the LLG equations of motion, following the method of Ref. [7], which satisfies the time averaged expectation value relations

$$\langle \mathbf{B}_{\rm th}(t) \rangle = 0, \tag{S15}$$

$$\langle B^i_{\rm th}(t) B^j_{\rm th}(t') \rangle = \frac{2k_{\rm B}Tg}{M_{\rm s}\gamma V \delta t} \delta_{tt'} \delta^{ij}, \tag{S16}$$

where γ is the gyromagnetic ratio, g is the dimensionless Gilbert damping parameter, $M_{\rm s}$ is the saturation magnetization, V is the spatial volume of a magnetic unit cell, and δt is the time step size of the numerical integration.

We numerically integrate a model consisting of three hexagons sharing three edges, with each edge corresponding to one spin $\mathbf{S}_i(t)$, for a total of 15 spins. In order to model the dynamics of a classical version of a $Q = 3$ excitation, for the initial state, the three central spins are oriented outward, with the rest of the spins arranged in an energy minimizing orientation along their respective axes. For ferromagnetic coupling, this central $Q = 3$ excitation state corresponds to an unstable fixed point of the LLG equations of motions. For sufficiently small temperatures, corresponding to small thermal fluctuations $\mathbf{B}_{\rm th}(t)$, this model predicts an identical relaxation time for a given arrangement over a broad range of temperatures. However, we find that when the root mean square of $\mathbf{B}_{\rm th}(t)$ becomes comparable to the anisotropy field $\mathbf{B}_{\rm an} \propto K_1$, the relaxation time begins to decrease monotonically as a function of temperature, as shown in Fig. 4a.

The existence of this anisotropy dependent crossover allows us to estimate a thermalization crossover temperature $T_{\rm c}$ for the classical model. The values used for this estimate are $\delta t = 10^{-11}$ s, $g = 0.2$, $M_{\rm s} = 6.6 \times 10^5$ A/m, and $V = 264\,{\rm nm}^3$. The resulting crossover temperature estimate is on the order of $T_{\rm c} \sim 20$ K, which is much too small to be consistent with the experimental results. The substantially better agreement of the experiment with quantum modeling provides evidence for our hypothesis on the robust quantum mechanical nature of the ultra-small artificial magnetic honeycomb lattice.

We again note that an effective value of K_1 can be difficult to estimate due to its emergent nature and due to the fact that the shape anisotropy intrinsically depends on the geometry of the material, which in this case is highly nontrivial. In spite of this, we believe that our theoretical model supports our conclusions of the dominant physics at play.

III. SUPPLEMENTARY REFERENCES

1 Pappas, C., Ehlers, G. & Mezei, F. *Neutron Scattering from Magnetic Materials Ch. 11.* (Elsevier, Amsterdam, 2006).

2 Ehlers, G., Stewart, J. R., Wildes, A. R., Deen, P. P. & Andersen, K. H. Generalization of the classical xyz-polarization analysis technique to out-of-plane and inelastic scattering. *Rev. Sci. Instrum.* **84**, 093901–093908 (2013).

3 Mezei, F. *Neutron Spin Echo Spectroscopy: Basics, Trends and Applications.* (Springer Germany, Berlin, 2003).

4 Zolnierczuk, P. A. et al. Efficient data extraction from neutron time-of-flight spin-echo raw data. *J. Appl. Cryst.* **52**, 1022–1034 (2019).

5 Chern, G. W. & Tchernyshyov, O. Magnetic charge and ordering in kagome spin ice. *Phil. Trans. R. Soc. A* **370**, 5718–5737 (2012).

6 Adler, S. B., Smith, J. W. & Reimer, J. A. Dynamic Monte Carlo simulation of spin-lattice relaxation of quadrupolar nuclei in solids. Oxygen-17 in yttria-doped ceria. *J. Chem. Phys.* **98**, 7613–7620 (1993).

7 Leliaert, J. et al. Adaptively time stepping the stochastic Landau-Lifshitz-Gilbert equation at nonzero temperature: Implementation and validation in MuMax3. *AIP Adv.* **7**, 125010–125022 (2017).
